# GMPPB-congenital disorders of glycosylation associate with decreased enzymatic activity of GMPPB

**DOI:** 10.1186/s43556-021-00027-2

**Published:** 2021-05-10

**Authors:** Zhe Liu, Yan Wang, Fan Yang, Qin Yang, Xianming Mo, Ezra Burstein, Da Jia, Xiao-tang Cai, Yingfeng Tu

**Affiliations:** 1 Key Laboratory of Birth Defects and Related Diseases of Women and Children, Department of Paediatrics, State Key Laboratory of Biotherapy, West China Second University Hospital, Sichuan University, Chengdu, 610041 China; 2 Department of Pediatric Surgery and Laboratory of Stem Cell Biology, State Key Laboratory of Biotherapy, West China Hospital, Sichuan University, Chengdu, 610041 China; 3 Department of Internal Medicine, University of Texas Southwestern Medical Center, Dallas, TX 75390 USA

**Keywords:** Congenital disorders of glycosylation, GMPPB, Enzymatic activity, Zebrafish model

## Abstract

The congenital disorders of glycosylation (CDG) are a family of metabolic diseases in which glycosylation of proteins or lipids is deficient. GDP-mannose pyrophosphorylase B (GMPPB) mutations lead to CDG, characterized by neurological and muscular defects. However, the genotype-phenotype correlation remains elusive, limiting our understanding of the underlying mechanism and development of therapeutic strategy. Here, we report a case of an individual presenting congenital muscular dystrophy with cerebellar involvement, who presents two heterozygous GMPPB mutations (V111G and G214S). The V111G mutation significantly decreases GMPPB’s enzymatic activity. By measuring enzymatic activities of 17 reported GMPPB mutants identified in patients diagnosed with GMPPB-CDG, we discover that all tested GMPPB variants exhibit significantly decreased enzymatic activity. Using a zebrafish model, we find that Gmppb is required for neuronal and muscle development, and further demonstrate that enzymatic activity of GMPPB mutants correlates with muscular and neuronal phenotypes in zebrafish. Taken together, our findings discover the importance of GMPPB enzymatic activity for the pathogenesis of GMPPB-CDG, and shed light for the development of additional indicators and therapeutic strategy.

**Supplementary Information:**

The online version contains supplementary material available at 10.1186/s43556-021-00027-2.

## Introduction

The congenital disorders of glycosylation (CDG), a heterogeneous category of disorders, lead to malfunction of multiple organs, especially the nervous system, muscles, and intestines in affected individuals [1–3]. Patients with CDG display a wide spectrum of clinical phenotypes, including but not limited to ataxia, seizures, cerebellar hypoplasia, liver diseases, limb-girdle muscular dystrophies (LGMD), and severe congenital muscular dystrophies (CMD) with eye and brain abnormalities [1, 2, 4, 5]. To date, mutations in more than 100 genes have been shown to be responsible for CDG [1, 6, 7]. For instance, genetic mutations of phosphomannomutase 2 (PMM2), the enzyme responsible for the conversion of mannose-6-phosphate into mannose-1-phosphate, lead to the most common subtype, PMM2-CDG [8, 9]. By far, treatment is not available for most of these disorders. One of the genes that have been recently linked with CDG is guanosine diphosphate mannose (GDP-mannose) pyrophosphorylase B (GMPPB) [10–12]. GMPPB catalyzes the formation of GDP-mannose, using mannose-1-phosphate and GTP as substrates, together with GDP-mannose pyrophosphorylase A (GMPPA). GDP-mannose acts as a sugar donor and participates in four glycosylation pathways, including N-glycosylation, O-mannosylation, C-mannosylation, and glycosylphosphatidylinositol (GPI)-anchor formation [11].

To date, more than 50 mutations in *GMPPB* gene have been identified in affected individuals. Most of the variants are missense, and the remaining mutations include nonsense, frame shift, and splicing. Interestingly, GMPPB mutations-associated phenotypic spectrum ranges from LGMD, to congenital myasthenic syndrome (CMS), to severe CMD with eye and brain symptoms [10–18]. However, there is no clear genotype-phenotype correlation. The mechanism underlying GMPPB mutations-related disorders remains elusive, and no effective therapeutic strategies are available. Given the essential role of GMPPB in catalyzing the formation of GDP-mannose, we hypothesize that the enzymatic activity of GMPPB mutants may correlate with the development of GMPPB-CDG.

In the present study, we report two novel GMPPB mutations (V111G/G214S) in one patient presenting CMD with cerebellar involvement. The V111G mutant displays significantly decreased enzymatic activity. By measuring enzymatic activity of 17 reported GMPPB variants identified in GMPPB-CDG patients, we find that enzymatic activities of these GMPPB variants are significantly impaired, indicating the involvement of compromised activity in the pathogenesis of GMPPB-CDG. This conclusion is further supported by studies using a zebrafish model. Altogether, our study illustrates that impaired GMPPB enzymatic activity might be a consensus causative factor for GMPPB-CDG.

## Results

### Identification of novel GMPPB mutations in a patient affected by CMD with cerebellar involvement

**Fig. 1:**
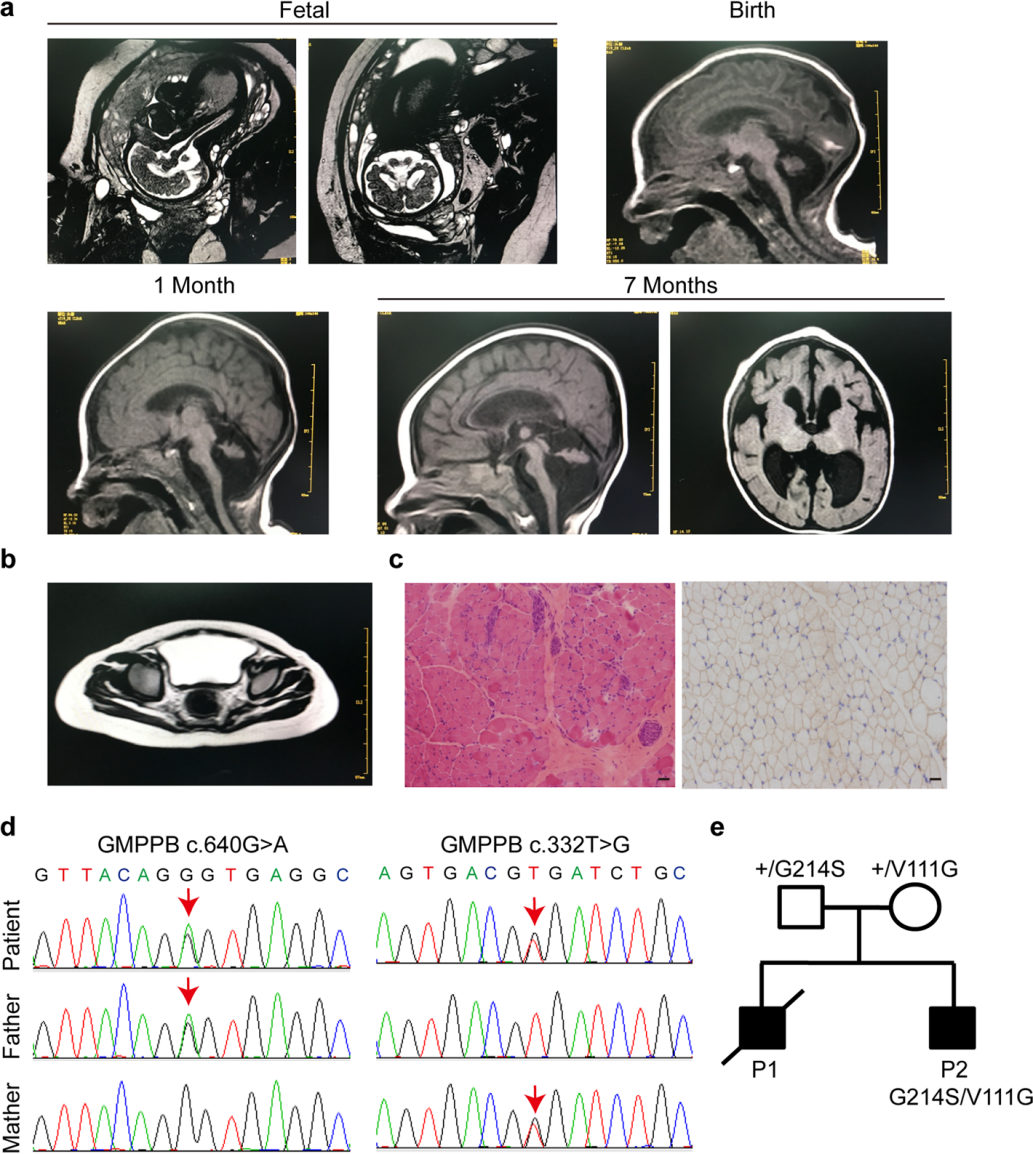
Clinical and genetic characterization. **a** Brain MRI of the patient at fetal 34 weeks, birth, 1 month, and 7 months, revealing evidence of cerebellar hypoplasia. **b** Muscle MRI in the right gluteus maximus muscle at 14 months. **c** Muscle biopsy from gastrocnemius at 14 months. The diameter of muscle fibers range between 5 and 50 μm, with occasional degenerated and regenerated muscle fibers. Scale bar: 50 μm **d** Sequence chromatograms of GMPPB gene of the patient. Variations and corresponding WT sequences are shown. The patient carries two missense mutations of GMPPB gene (arrows), inherited from his mother (c.332 T > G) and father (c.640G > A), respectively. **e** Pedigrees

The patient was a 1-year old Chinese male, who was diagnosed with CMD with cerebellar involvement (CMD-CRB). His brain showed smaller size of cerebellar hemisphere and enlarged bilateral lateral ventricles, detected by MRI at his fetal stage (34 week), which indicated a possibility of cerebellar hypoplasia (Fig. 1a). Postnatal brain MRI result also revealed an abnormal development of cerebellum (Fig. 1a). The patient also presented with muscle weakness in upper and lower limbs as early as the age of 1 month. He gradually displayed more behavioral problems, including difficulties in raising his head and crawling. Muscle MRI showed edema of gluteus maximus (Fig. 1b). Muscle biopsy indicated chronic myopathy (Fig. 1c). During following-up examination, elevated creatine kinase (CK) was recorded, which fluctuated between 391 and 898 U/l (55-170 U/l) (Table 1), consistent with muscle defects.

**Table 1:** Clinical features of the patient

Age	Birth	1 Month	3 Months	7 Months	14 Months	18 Months
Head circumference (cm)	31	31.5	33	35	39	41
Weight (g)	1900	1800	3000	4000	7000	9000
Fontanel	1*1 cm	0.3*0.3 cm	0.3*0.3 cm	closed	closed	closed
Tic	none	sporadic	4–5 times/day	20+ times/day	10+ times/day	10+ times/day
Creatine kinase (U/l)	–	898 U/l	–	391 U/l	674 U/l	–
Brain MRI	decreased vermis, widened posterior fossa, enlarged bilateral ventricles	decreased cerebellar hemispheres and vermis, widened posterior fossa, enlarged bilateral lateral ventricles	–	delayed myelination, decreased cerebellar hemispheres and vermis, widened posterior fossa, enlarged bilateral lateral ventricles and third ventricle	–	–
Muscle MRI	–	–	–	–	oedema of the right gluteus Maximus muscle	–
Muscle biopsy	–	–	–	–	Chronic myopathy	–
Presenting signs and symptoms	–	decreased tone	Poor head control, decreased tone	difficulty in crawling and turning over, dysphagia, decreased tone	no eye contact, gastric tube feeding, difficulty in crawling and turning over, decreased tone, less active limbs	no eye contact, gastric tube feeding, contractures in the knuckles of both hands, less active limbs

Using whole exome sequencing, two compound heterozygous mutations in GMPPB gene (c.332 T > G (p.Val111Gly) and c.640G > A (p.Gly214Ser)) (Fig. 1d) were identified in the patient. The mutations were inherited from his mother and father, respectively, who displayed no obvious abnormality (Fig. 1e). Furthermore, the first fetus of the same parents was also found to have cerebellar atrophy by fetal brain MRI, and the parents chose to abort in accordance with their wills. (Fig. 1e, P2).

### CDG-associated GMPPB variants exhibit significantly decreased enzymatic activity

**Fig. 2:**
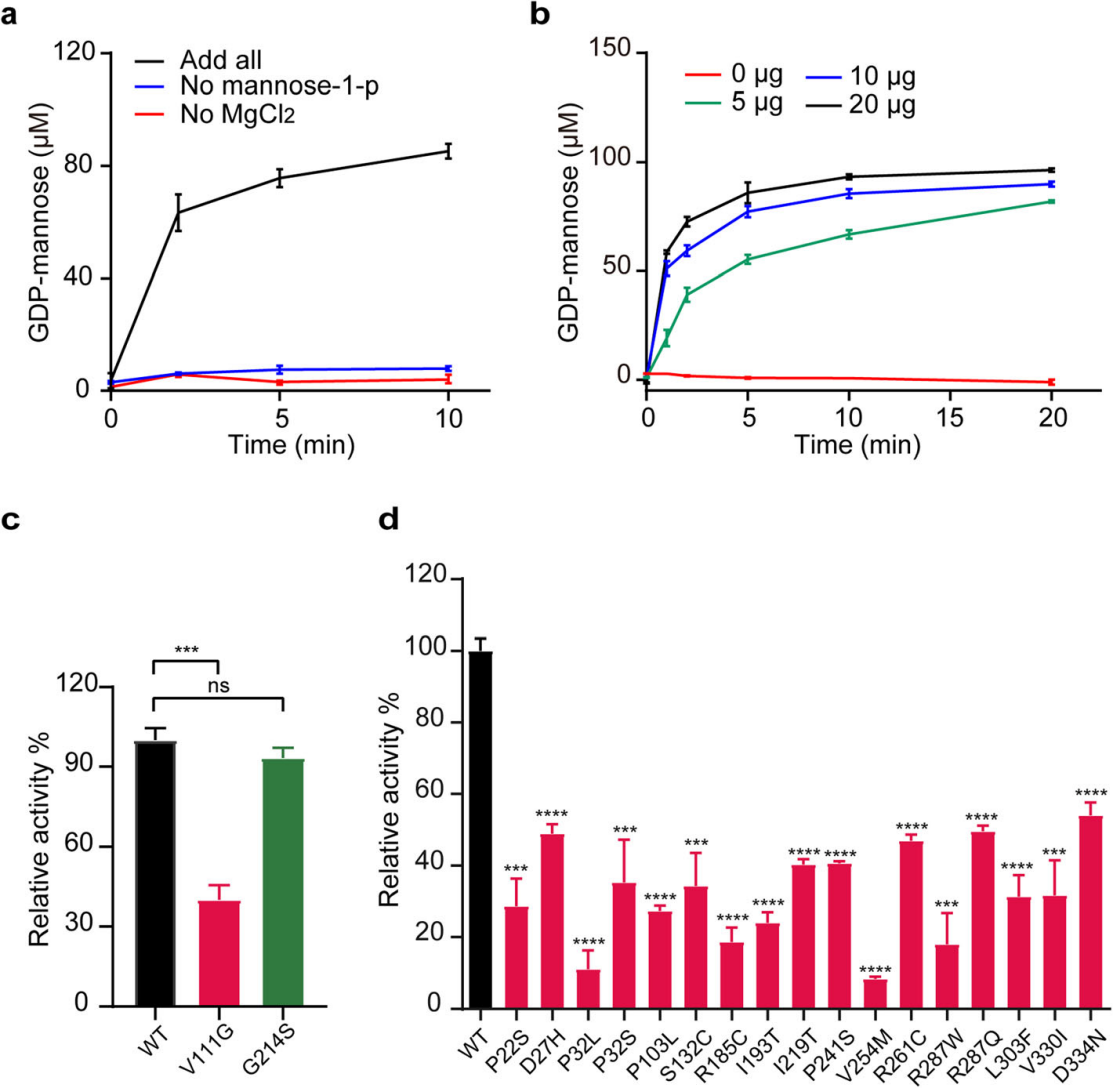
GMPPB mutants exhibit significantly decreased enzymatic activity. **a-b** Validation of the designed system for evaluating GMPPB’s enzymatic activity. Absence of Mg^2+^ or substrate mannose-1-p leads to no detectable formation of GDP-mannose **(a)**. The time course of GDP-man production catalyzed by variant doses of GMPPB **(b)**. 0 μg, 5 μg, 10 μg or 20 μg purified GMPPB was used to catalyze the reaction, which was terminated at the indicated time. The reaction mixtures were subjected to Q-ion exchange chromatography, and the amount of GDP-mannose was determined. **c** V111G mutation causes significantly decreased activity of GMPPB. Purified GMPPB WT, GMPPB V111G, or GMPPB G214S were subjected to activity detection. Compared to the WT, V111G mutation caused significant reduction of GMPPB activity, whereas GMPPB G214 variant retained activity comparable to WT. **d** Disease-associated GMPPB mutation results in significantly decreased enzymatic activity. 17 reported GMPPB mutants identified in patients were expressed in E.coli and subjected to activity detection. All the tested mutants exhibited significantly compromised activity relative to GMPPB WT. Mean ± SD, ****p < 0.0001; ***p < 0.001; **p < 0.01; *p < 0.05. *p* values were calculated using one-way ANOVA, Tukey’s multiple comparisons test

To test whether enzymatic activity of GMPPB variants could be a determinant for the development of GMPPB-CDG, we developed an assay to measure enzymatic activity of GMPPB. GMPPB catalyzes the conversion of GTP and mannose-1-p to GDP-mannose and pyrophosphoric acid. Q-ion exchange chromatography was used to separate GDP-mannose and GTP, due to their difference in negative charge, and the amount of GDP-mannose was used to assess activity of GMPPB. Consistent with essential roles of Mg^2+^ or mannose-1-p in the reaction [11, 19], GDP-mannose couldn’t be produced in the absence of Mg^2+^ or mannose-1-p (Fig. 2a). As shown in Fig. 2b, the amount of GDP-mannose produced was nearly linear with time for the initial 5 min, and the reaction reached saturation within 20 min. Furthermore, GMPPB catalyzed the reaction in a dose-dependent manner (Fig. 2b). These results indicated that our assay is effective to evaluate GMPPB activity. We then sought to determine the effects of GMPPB mutations identified in our patient on its activity. Interestingly, the V111G mutant decreased enzymatic activity by ~ 60%, while the activity of G214S was comparable to that of GMPPB wild-type (WT) (Fig. 2c). We also examined the effects of the missense mutations on the subcellular localization of GMPPB. We found that V111G mutation caused increased nuclear localization of GMPPB, while G214S mutant seemed to lose nuclear localization (Supplementary Fig. 1).

Next, we selected 17 reported GMPPB missense mutants identified in patients, who displayed symptoms ranged from mild to severe conditions. These GMPPB variants were expressed in *E.coli*, and their enzymatic activity was measured. All the tested mutants displayed markedly decreased enzymatic activities, with P32L and V254M being the lowest (~ 10% of GMPPB WT) (Fig. 2d). These results indicated that impaired enzymatic activity of GMPPB might be a consensus causative factor for GMPPB-CDG.

### Gmppb is required for neuronal and muscle development in zebrafish

**Fig. 3:**
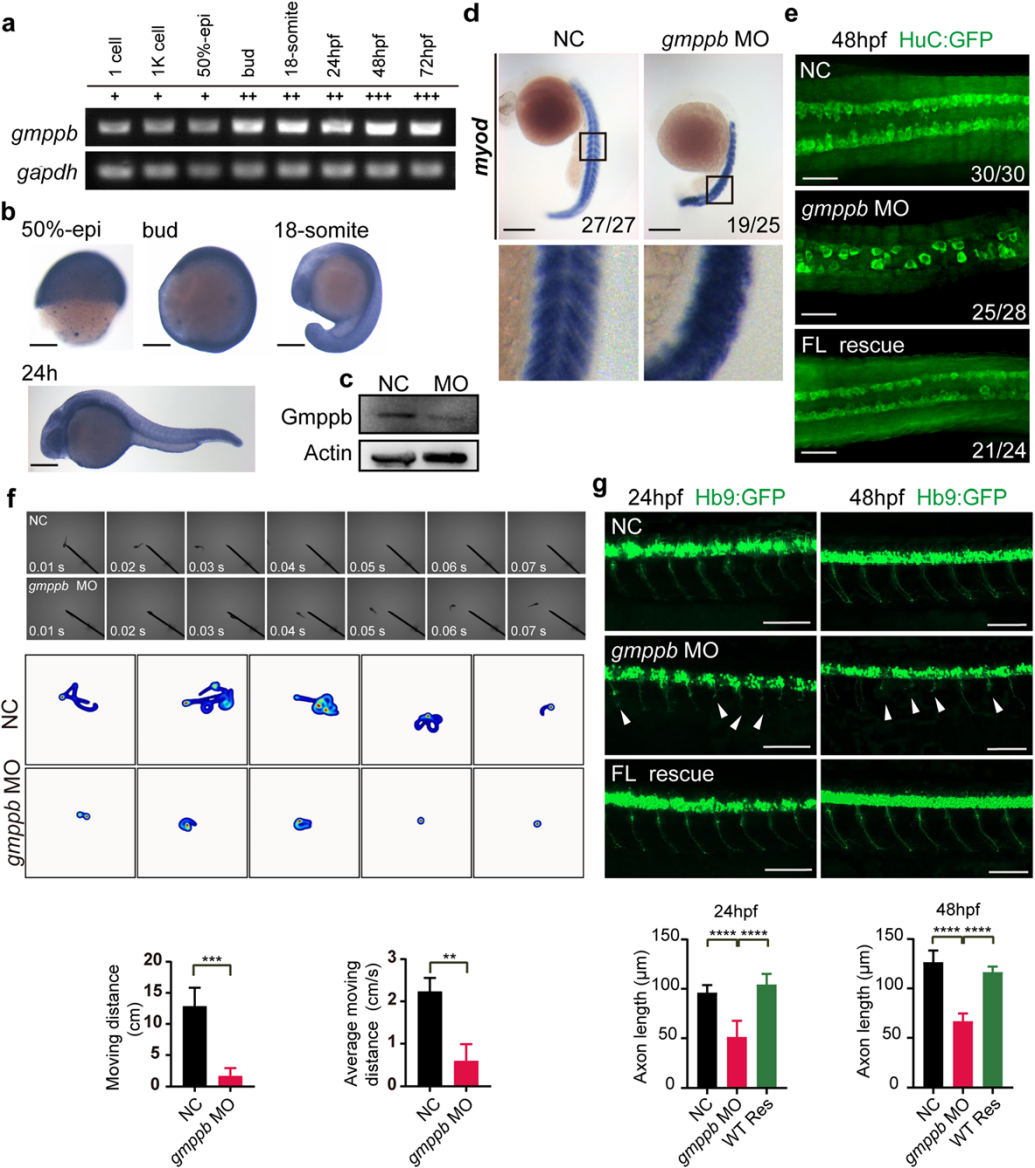
Gmppb knockdown(KD) in Zebrafish causes abnormal development of muscle and motor neurons, movement disability. **a** Temporal expression of zebrafish *gmppb* from fertilization to day 3, and *gapdh *was used as control. **b** WISH shows the spatial expression pattern of *gmppb* using a dig-labeled antisense probe. *gmppb* was ubiquitously expressed in zebrafish embryos from 50%-epi to 24hpf. Scale bar: 250 μm. **c** Immunoblot of entire zebrafish tissue extracts indicates that *gmppb* MO injection effectively decreased Gmppb protein level. MO injection was performed at the one cell stage. Actin was used as a loading control. **d** Expression of *myod* in zebra fish, with or without *gmppb* MO injection, determined by WISH. The black rectangles label the position of enlarged views (bottom). Scale bar: 250 μm. **e** Gmppb KD results in decreased HuC (green) expression. Tg [HuC: GFP] transgenic zebrafish was injected with control MO (NC), or *gmppb* MO alone or together with mRNA encoding GMPPB. HuC expression was observed at 48 hpf. Scale bar: 100 μm**f** *gmppb* mutant larvae display decreased motor ability. Representative images and summarized movement path of zebrafish injected with control MO (NC) or *gmppb* MO. (Upper). Bar graph summarizes the moving distance and moving speed of zebrafish injected with control MO (NC) or *gmppb* MO (Lower). Motor ability was monitored in 2 dpf larvae. **g**. GMPPB is required for motor neuronal development in zebra fish. Tg [hb9: GFP]^ml2^ transgenic zebrafish was injected with control MO (NC), or *gmppb* MO alone or together with mRNA encoding GMPPB at one-cell stage. Top: morphology of CaP axons was observed at 24 and 48hpf; bottom: statistical results of the length of CaP axons. Scale bar: 100 μm. Mean ± SD, ****p < 0.0001; ***p < 0.001; **p < 0.01; *p < 0.05. *p* values were calculated using one-way ANOVA, Tukey’s multiple comparisons test

To determine the functions of GMPPB in development, we chose zebrafish as a model organism. Semi-quantitative RT-PCR indicated that *gmppb* could be detected at as early as the one-cell stage. Expression of *gmppb* gradually increased at bud stage, and peaked at 48 h post fertilization (hpf), suggesting a role in early embryonic development. (Fig. 3a). Whole mount in situ hybridization (WISH) showed that spatial expression of *gmppb* was ubiquitous during embryonic development (Fig. 3b). Using *gmppb*-specific morpholino (MO), we were able to successfully deplete most Gmppb protein in *gmppb*-MO-injected embryos (Fig. 3c). Consistent with the previous study [13], we also observed sparse and disordered muscle fibers in *gmppb-*MO-injected embryos (Fig. 3d and Supplementary Fig. 2). Furthermore, immunofluorescence assay revealed that the level of HuC (*elavl3*), an early marker of pan-neuronal cells, was remarkably decreased in the MO embryos. Importantly, the decrease of HuC could be effectively rescued by co-injection of mRNA encoding human GMPPB WT (Fig. 3e). Analogous to patients with GMPPB-CDG, *gmppb* MO-injected zebrafish exhibited remarkably decreased motor ability, as shown by reduced moving distance and moving speed (Fig. 3f).

Given muscle defects and movement disability of patients with GMPPB-CDG and of *gmppb *MO-injected embryos, we suspected that Gmppb might be involved in development of motor neurons. Thus, we chose Tg [hb9: GFP]^ml2^ transgenic zebrafish to examine morphology of CaP motor neurons [20, 21]. These neurons project ventrally in the middle of each spinal cord, and can be readily examined. Injection of *gmppb* MO led to shorter CaP axons at 24 and 48 hpf, which were less than half of WT. Again, the axonal phenotype could be rescued to the control level by co-injection of mRNA encoding human GMPPB WT (Fig. 3g). Taken together, Gmppb is essential for neuronal and muscle development in zebra fish.

### Enzymatic activity of GMPPB variants correlates with muscular and neuronal phenotypes in zebrafish

**Fig. 4:**
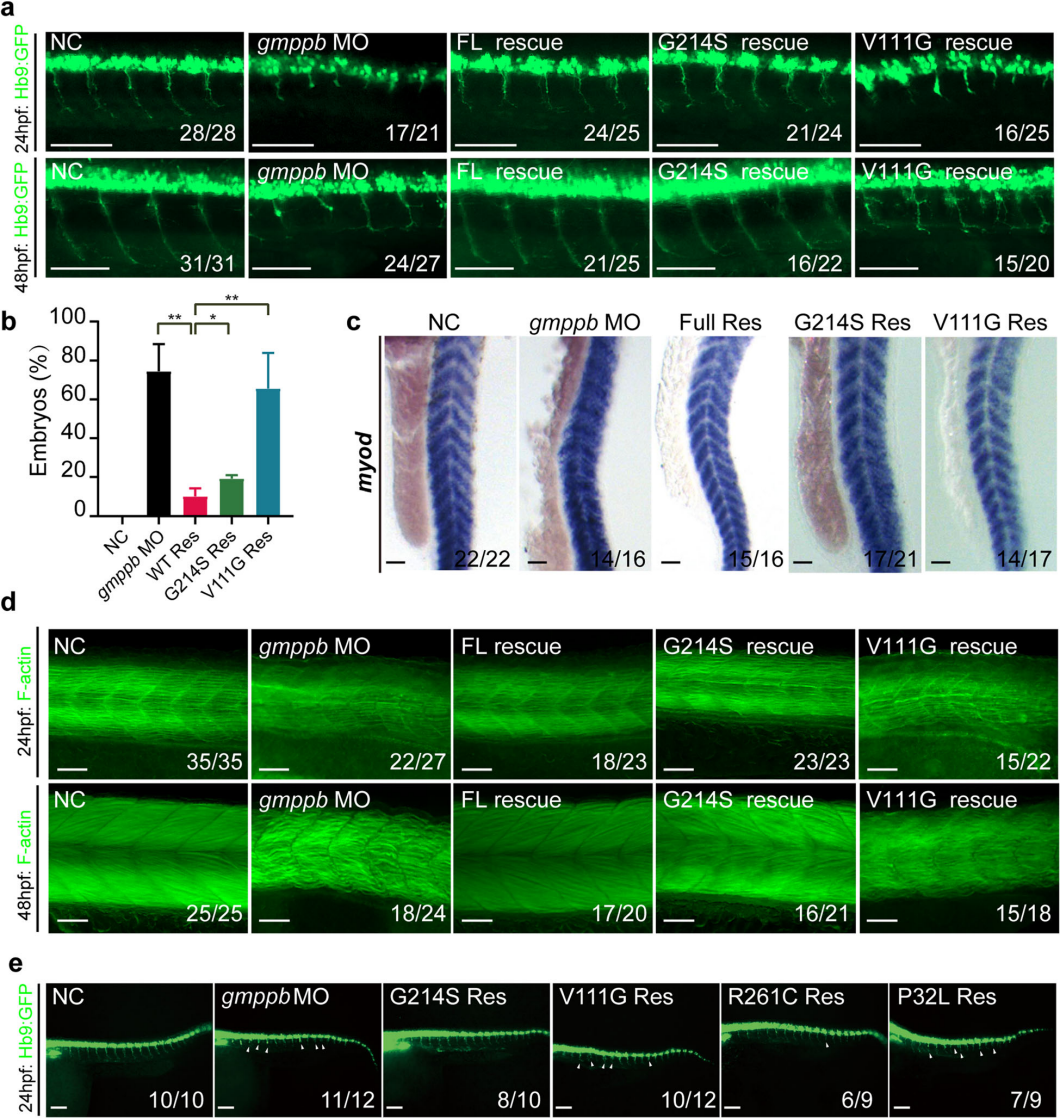
GMPPB activity is required for muscle and neuronal development (**a-b**) GMPPB V111G mutant with decreased activity fails to rescue axonal phenotype in *gmppb* MO-injected zebrafish. Morphology of CaP axons from embryos at 24 and 48 hpf that were injected with control MO (NC), or gmppb MO alone or together with mRNA encoding GMPPB WT or its mutants at one-cell stage of the Tg [hb9: GFP]^ml2^ transgenic zebrafish embryos. Scale bar: 100 μm (**a**). Statistical results of the embryos with abnormally branched axons (**b**). **c-d** GMPPB V111G mutant fails to rescue muscle defects caused by Gmppb KD. Expression of *myod* in zebra fish injected with control MO (NC), or *gmppb* MO alone or together with mRNA encoding GMPPB WT or its mutants. Scale bar: 100 μm (**c**). Phalloidin staining of filamentous actin (green) in zebra fish injected with control MO (NC), or *gmppb* MO alone or together with mRNA encoding GMPPB WT or its mutants at one-cell stage. Scale bar: 100 μm (**d**). **e** The enzymatic activity of GMPPB mutant positively correlates its ability in rescuing axonal phenotype. Morphology of CaP axons from embryos at 24 and 48 hpf that were injected with control MO (NC), or *gmppb* MO alone or together with different mRNA at one-cell stage of the Tg [hb9: GFP]^ml2^ transgenic zebrafish embryos. Scale bar: 80 μm. Mean ± SD, ****p < 0.0001; ***p < 0.001; **p < 0.01; *p < 0.05. *p* values were calculated using one-way ANOVA, Tukey’s multiple comparisons test

Finally, we sought to determine whether enzymatic activity of GMPPB variant correlates with phenotypes in zebrafish. *gmppb* MO was co-injected with mRNA encoding human GMPPB WT, V111G, or G214S. G214S, which displayed enzymatic activity similar to GMPPB WT, could rescue axonal phenotypes at 24 and 48 hpf. In contrast, V111G, which exhibited significantly decreased activity, failed to do so (Fig. 4a and b). Furthermore, GMPPB WT and G214S, but not V111G, were able to restore the muscular phenotypes of MO-injected embryos, which displayed sparse and disordered muscle fibers (Fig. 4c, d and Supplementary Fig. 3). Altogether, these data indicated that enzymatic activity of GMPPB is critical for muscular and neuronal development.

To corroborate our conclusion, we chose two additional GMPPB mutations, R261C and P32L, which displayed 50% and 10% enzymatic activity of WT, respectively. Co-injection of R261C partially restored decreased length of CaP neurons caused by GMPPB MO; in contrast, GMPPB P32L was unable to rescue (Fig. 4e). Thus, enzymatic activity of GMPPB might be a critical determinant for phenotypes of zebrafish.

## Discussion

So far, more than 50 different GMPPB mutations have been reported. The clinical spectrum of GMPPB-CDG spans from CMS, LGMD to CMD with or without brain abnormalities [10, 13, 14, 18, 22]. However, the mechanism underlying this broad phenotypic spectrum remains unknown. In the present study, we report two novel GMPPB mutations in a patient diagnosed with CMD. We identify enzymatic activity of GMPPB as a key determinant of GMPPB-CDG development. And we find that enzymatic activity of GMPPB variant correlates its ability in rescuing muscular and neuronal phenotypes in zebrafish. Our study suggests that the cellular level of GDP-mannose could correlate with patients’ symptoms.

To evaluate the effects of GMPPB mutations on its enzymatic activity, WT GMPPB and its mutants were expressed and further purified as MBP-fusion protein. MBP tag (~ 42 kDa) is a relatively big tag and might affect protein function [23]. We tried to remove the MBP tag of the fusion protein, which unfortunately produced insoluble protein. We also expressed GMPPB with a His6-tag, but did not obtain soluble protein. As the MBP-tagged GMPPB catalyzes the formation of GDP-mannose in time and concentration dependent manners, it is the best available approach to compare the activity of wild-type and mutants proteins.

GMPPB is comprised of two functional domains: N-terminal catalytic domain (residues 1–250) and a left-handed parallel beta-helix domain (residues 251–360) [18]. Whereas disease-causing mutations are found through the entire GMPPB protein, mutations within its catalytic domain impair activity more severely than those in the LbH domain. Consequently, individuals carrying these variants display more severe symptoms. How do GMPPB mutations affect its functions? Previous studies suggested that some mutants, such as D334N, P22S, P32L, R287Q, V330I, V254M, R287W, I268T, and G354S, altered subcellular localization of GMPPB [10, 13, 18]. And V111G and G214S mutations identified in our patient also altered subcellular localization of GMPPB (Supplementary Fig. 1). Furthermore, some mutations, including V254M,. R287W, I268T, and G354S, impaired GMPPB protein level [10, 18]. Interestingly, reduced protein levels of I268T and G354S could be rescued by lysosomal inhibitors, indicating the involvement of autophagy-lysosomal pathway in destabilization of GMPPB [18]. The disease-causing mechanisms of other mutations warrant further investigation.

Significant efforts have been spent in the past in order to understand the underlying mechanism of each subtype of CDG and to develop specific treatment strategy. For instance, prenatal supplementation of mannose successfully overcomes embryonic lethality of Pmm2 mice, and has been proposed to treat PMM2-CDG [24, 25]. However, mannose supplementation failed to rescue lethality in a mice bearing the R137H/F115L genotype (corresponding to the two most prevalent mutations in patients with PMM2-CDG: R141H and F119L). Both PMM2-CDG mouse model and PMM2-CDG patient-derived fibroblasts were reported to display decreased PMM activity, GDP-mannose and total cellular protein glycosylation [26]. And supplementation of mannose, the most upstream substrate, might not be sufficient to compensate the severely impaired PMM2 activity, contributing to its inability to rescue lethality in PMM2 ^R137H/F115L^ mice. During our efforts to search for therapeutic regimen for GMPPB-CDG, we found that supplementation of GDP-mannose effectively rescues the phenotypes in zebrafish model (unpublished data in our complementary paper). However, glucose or mannose, the most upstream substrate, failed to rescue muscle defects and abnormal development of motor neurons observed in *gmppb* MO-injected embryos. Our study demonstrates GMPPB’s enzymatic activity as a potential causative factor for GMPPB-CDG, and suggests that supplementation of GDP-mannose or its analogs may represent a potential therapeutic strategy for GMPPB-CDG.

## Materials and methods

### Genetic analysis

Genomic DNA was extracted from blood using standard procedures. Whole exome sequencing was performed for the patient as previously described [27]. The obtained sequences were mapped to human genome build hg19.Variants were called using LifeScopeTM 2.5 (Life Technologies). Variants were then filtered using ANNOVAR [28] against the ENCODE GencodeV14 [29].

### Muscle pathology

We performed muscle biopsy on the right gastrocnemius in the patient at 14 months. The muscle tissue was frozen and cut at 7 μm. Sections were stained with Hematoxylin and Eosin (HE). Sections were also immunostained with the antibody against dystrophin with standard procedures [18].

### Protein expression and purification

Human GMPPB cDNA was cloned into a modified pMAL vector with an N-terminal MBP tag. GMPPB mutants were generated by site-directed mutagenesis. Expression of GMPPB WT or mutants was induced in BL21 (DE3) cells by 0.5 mM isopropyl β-D-1-thiogalactopyranoside (IPTG) at 16 °C for 12 h. Cells were harvested, and re-suspended with the lysis buffer (20 mM Tris-HCl, pH 8.0, 200 mM NaCl, 1 mM DTT, 1% PMSF). Cells were broken by high-pressure homogenizer, and subjected to centrifugation. Proteins were purified with amylose-affinity resin, and eluted into Buffer 1 (20 mM Tris-HCl, pH 8.0, 200 mM NaCl, 1 mM DTT) supplemented with 1% (w/v) maltose.

### Enzymatic activity assay

For enzymatic activity assays, purified GMPPB protein (10 μg) was mixed with 0.5 mM Man-1-P, 0.25 mM GTP, 50 mM Tris, pH 7.5, and 1 mM MgCl_2_. The reaction was carried out at 37 °C for 5 min, and terminated by the addition of NaOH (20 μl, 10 M). After termination, the reaction mixtures were subjected to Q-ion exchange chromatography, which separated GDP-mannose from GTP. The eluted positions of GDP-mannose and GTP were also confirmed using pure chemicals. The amount of GDP-mannose and GTP were determined by integrating their absorption at 260 nm. All experiments were conducted at least three times.

### Immunofluorescence

C2C12 transfected with indicated constructs were fixed in 4% paraformaldehyde for 15 min at room temperature, and then permeabilized with 0.25% Triton X-100 for 15 min. The permeabilized cells were blocked with 5% BSA for 1 h at room temperature and incubated with primary antibodies against HA (proteintech, 66,006–2-Ig) at 4 °C overnight. Cells were then incubated with Alexa Fluor 546-labeled goat-anti-mouse secondary antibody for 1 h. The cells were further counterstained with DAPI to visualize nucleus. Images were taken with equal exposure time.

### Zebrafish maintenance

All zebrafish experiments were performed according to standard procedures. Adult fish and embryos were raised at 27.5–28.5 °C in the Aquatic Ecosystems. The lines used in this study include: AB strain (wild-type), Tg[hb9:GFP]^ml2^ transgenic lines and Tg[HuC:GFP] strain. All experimental protocols were proved by the Animal Ethics Committee, West China Second University Hospital.

### Antisense RNA probes and mRNAs

Antisense RNA probes for in situ hybridization were synthesized in vitro using the Riboprobe system kit (Promega). DNA sequences encoding human GMPPB WT or mutants were cloned into a pcDNA3.1+ vector, and were used as the template for Capped mRNAs synthesis using T7 mMESSAGE mMACHINE kit (Ambion).

### Zebrafish knockdown and mRNA injection

To knock down zebrafish *gmppb*, we chose a morpholino oligonucleotide (MO) (sequence: 5′-GGACCAGCTGAAAACAGAAACAGAT-3′), which was reported to efficiently deplete Gmppb [13]. Control MO is a standard mismatched control. MO and mRNAs were injected into the yolk and the cell at the stage of one cell, with 5 ng of MO and/or 200 pg of mRNA per injection.

### Total RNA isolation and semi-quantitative RT-PCR

RNA isolation and Semi-quantitative RT-PCR was carried out as previously described [30]. Fifty zebrafish embryos at indicated time point were collected for each sample. The embryos were grounded, and tissue debris was removed. Total RNA was isolated by RNeasy Plant Mini Kit (FOREGRNE), and then used to synthesize cDNA with the Prime Script Reverse-transcription PCR kit (TaKaRa DRR014A). The resultant cDNA was used as a template for RT-PCR, performed with the Real Master Mix Kit (Roche).

### Protein extraction and Western blot

Zebrafish embryos injected with control or *gmppb* MO were collected at the stage of 24 h and subjected to protein extraction. Briefly, after removal of oolemma and yolk, the embryos were lysed with RIPA buffer supplemented with 1% protease inhibitor mixture (Bimake, B14001) for 30 min and sonicated at 4 °C. After incubating on ice for 15 min, the lysate were centrifuged at 10000 rpm for 30 min at 4 °C. The supernatant were collected and protein concentration was measured by the bicinchoninic acid method. The supernatant were boiled in loading buffer and subjected to western blot analysis with the indicated antibodies (GMPPB, proteintech 15,094–1-AP; actin, Abclonal, AC026).

### Motor ability

Larval motor ability was measured on the second day of development (5 larva per group). Single larva was placed in a 10-cm dish, stimulated at the head by a tip of syringe, and its movement path was recorded. Data were analyzed using EthoVision software (Noldus, Inc.).

### Whole-mount in situ hybridization (WISH)

WISH analysis was performed as previously described [31]. In brief, samples at 24 hpf and 48 hpf were dechorionated, fixed with 4% paraformaldehyde (PFA), dehydrated in methanol (30% to 100%) overnight, and rehydrated with methanol (100% to 30%). Zebra fish embryos were incubated with proteinase K (1 mg/mL, Promega) for 20 min at room temperature, and then incubated with digoxigenin (DIG)-labeled antisense RNAs (*gmppb*, *myod*) at 65 °C overnight. After the probes were removed, the samples were incubated with alkaline phosphatase (AP)-conjugated anti digoxigenin antibody (Roche, 11,093,274,910, 1:2000) at 4 °C overnight. After washing, the samples were subjected to NBT/BCIP (Roche) staining according to the manufacturer’s instructions.

### Whole-mount immunofluorescence

Immunofluorescence was performed as previously described [20]. Briefly, zebrafish embryos were fixed with 4% PFA at 4 °C overnight, washed 10 times with PBS for 5 min each wash, incubated with acetone at − 20 °C for 10 min, and finally washed 10 times with PBS for 5 min each wash. Samples were blocked with 5% BSA containing 0.1% triton X-100, and incubated with phalloidin (Sigma, P5282, 1;2000) or Huc (Abcam, ab210554, 1:250) at 4 °C overnight. The embryos were then washed, and DAPI was used for nucleic acid staining. The embryos were examined by a Zeiss confocal laser-scanning microscope.

### Statistical analysis

All zebrafish and biochemical experiments were performed at least three times. Statistical analyses were performed using one-way ANOVA, Tukey’s multiple comparisons test incorporated in Prism 7 (GraphPad Software). ***P < 0.001, **P < 0.01, *P < 0.05, ns: not significant.

## Supplementary Information

**Additional file 1: Figure S1.** V111G and G214S mutations alter subcellular localization of GMPPB. C2C12 myoblasts were transfected with HA-GMPPB WT, HA-GMPPB V111G, HA-GMPPB G214S or HA-GMPPB D334N. Compared to WT GMPPB, V111G mutant exhibited increased localization in the nucleus, while G214S mutant seemed to lose nuclear localization. D334N, previously reported to form aggregates within the cytoplasm, was used as a control. Scale bar: 10 μm. **Figure S2.** GMPPB KD causes muscle defects in zebra fish. **(a)** Phalloidin staining (red) of filamentous actin in zebra fish injected with control MO (NC), or gmppb MO at one-cell stage. Scale bar: 250 μm **(b)** Phalloidin staining (green) on the cross-sections of 24 hpf embryos injected with control MO (NC), or gmppb MO at one-cell stage. Scale bar: 100 μm. **Figure S3.** GMPPB V111G mutant fails to rescue muscle defects in zebra fish caused by GMPPB KD. Phalloidin (green) and DAPI (blue) staining on the cross-sections of 48 hpf embryos injected with control MO (NC), or gmppb MO alone or together with mRNA encoding GMPPB WT or its mutants at one-cell stage. Scale bar: 100 μm.

## Data Availability

All data generated or analyzed during this study are included in this published article and its supplementary information files.

## Abbreviations

GDP-mannose: Guanosine diphosphate mannose
GMPPB: GDP-mannose pyrophosphorylase B
CDG: Congenital disorders of glycosylation
LGMD: Limb-girdle muscular dystrophies
CMD: Congenital muscular dystrophies
PMM2: Phosphomannomutase 2
GMPPA: GDP-mannose pyrophosphorylase A
GPI: Glycosylphosphatidylinositol
CMS: Congenital myasthenic syndrome
CMD-CRB: CMD with cerebellar involvement
CK: Creatine kinase
WISH: Whole mount in situ hybridization
MO: Morpholino
HE: Hematoxylin and Eosin
