# Supplementary material for: GMPPB-congenital disorders of glycosylation associate with decreased enzymatic activity of GMPPB
Source: Mol Biomed. 2021 May 10;2:13. doi: 10.1186/s43556-021-00027-2 (PMC8607393; doi:10.1186/s43556-021-00027-2)
Supplement: Supplementary file 1 — Additional file 1: Figure S1. V111G and G214S mutations alter subcellular localization of GMPPB. C2C12 myoblasts were transfected with HA-GMPPB WT, HA-GMPPB V111G, HA-GMPPB G214S or HA-GMPPB D334N. Compared to WT GMPPB, V111G mutant exhibited increased localization in the nucleus, while G214S mutant seemed to lose nuclear localization. D334N, previously reported to form aggregates within the cytoplasm, was used as a control. Scale bar: 10 μm. Figure S2. GMPPB KD causes muscle defects in zebra fish. (a) Phalloidin staining (red) of filamentous actin in zebra fish injected with control MO (NC), or gmppb MO at one-cell stage. Scale bar: 250 μm (b) Phalloidin staining (green) on the cross-sections of 24 hpf embryos injected with control MO (NC), or gmppb MO at one-cell stage. Scale bar: 100 μm. Figure S3. GMPPB V111G mutant fails to rescue muscle defects in zebra fish caused by GMPPB KD. Phalloidin (green) and DAPI (blue) staining on the cross-sections of 48 hpf embryos injected with control MO (NC), or gmppb MO alone or together with mRNA encoding GMPPB WT or its mutants at one-cell stage. Scale bar: 100 μm. [file 43556_2021_27_MOESM1_ESM.docx]

**Supplementary materials for**

**GMPPB- congenital disorders of glycosylation associate with decreased enzymatic activity of GMPPB**

**Zhe Liu ^1#^, Yan Wang^1#^, Fan Yang^1^, Qin Yang^1^, Xianming Mo^2^, Ezra Burstein^3^, Da Jia^1*^, Xiao-tang Cai^1*^, Yingfeng Tu^1*^**

^1^Key Laboratory of Birth Defects and Related Diseases of Women and Children, Department of Paediatrics, West China Second University Hospital, State Key Laboratory of Biotherapy, Sichuan University, Chengdu 610041, China

^2^Department of Pediatric Surgery and Laboratory of Stem Cell Biology, State Key Laboratory of Biotherapy, West China Hospital, Sichuan University, Chengdu, 610041, China

^3^Department of Internal Medicine, University of Texas Southwestern Medical Center, Dallas, TX 75390

# Equal contribution

^*^Corresponding author: D.J. ([Jiada@scu.edu.cn](mailto:Jiada@scu.edu.cn))

X.C. (cxt_1999@126.com)

Y.T. ([tuyingfeng2haha@sina.com](mailto:tuyingfeng2haha@sina.com))

This file includes:

Figs S1-S3


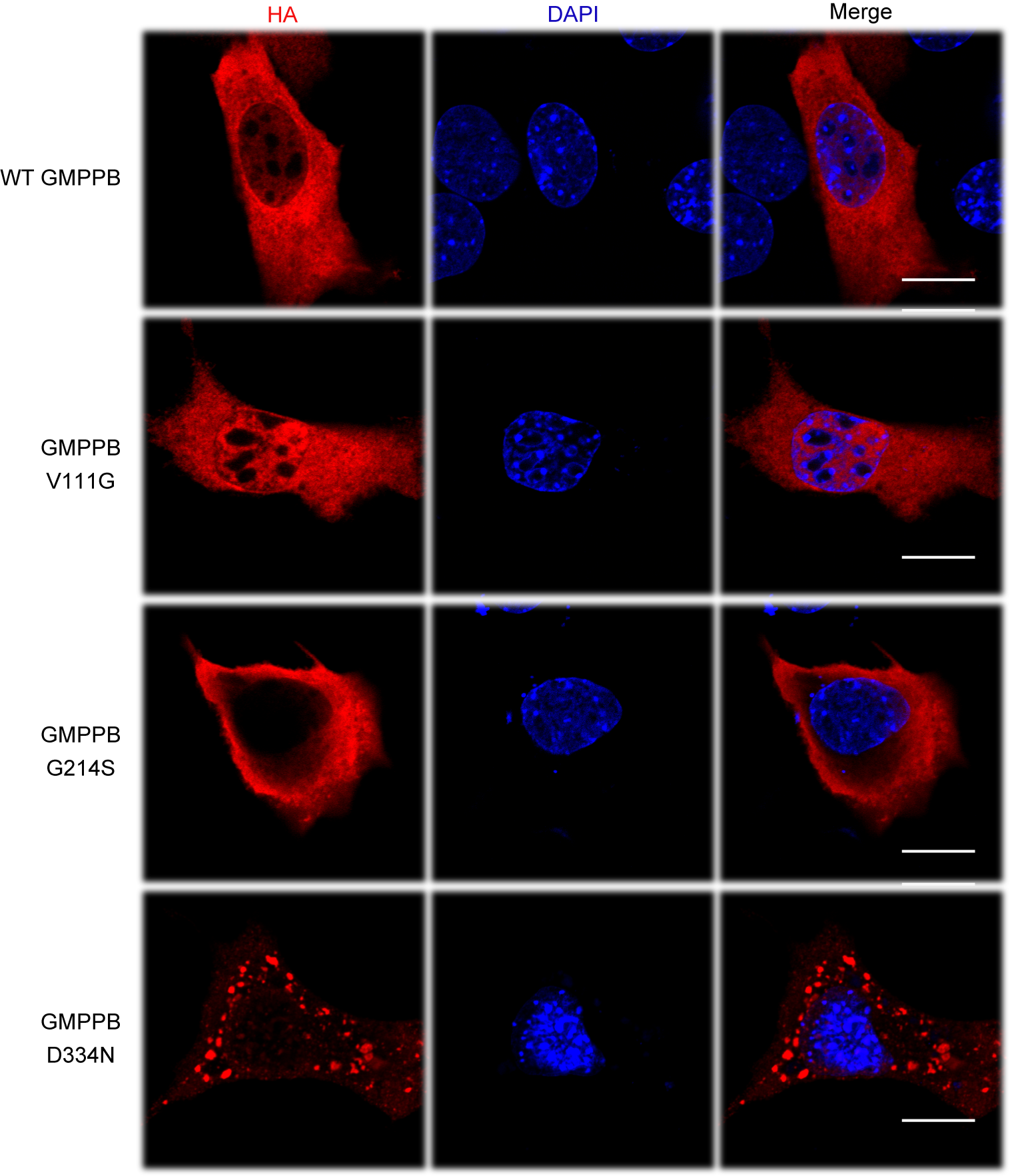


**Figure S1.** V111G and G214S mutations alter subcellular localization of GMPPB. C2C12 myoblasts were transfected with HA-GMPPB WT, HA-GMPPB V111G, HA-GMPPB G214S or HA-GMPPB D334N. Compared to WT GMPPB, V111G mutant exhibited increased localization in the nucleus, while G214S mutant seemed to lose nuclear localization. D334N, previously reported to form aggregates within the cytoplasm, was used as a control. Scale bar: 10 μm


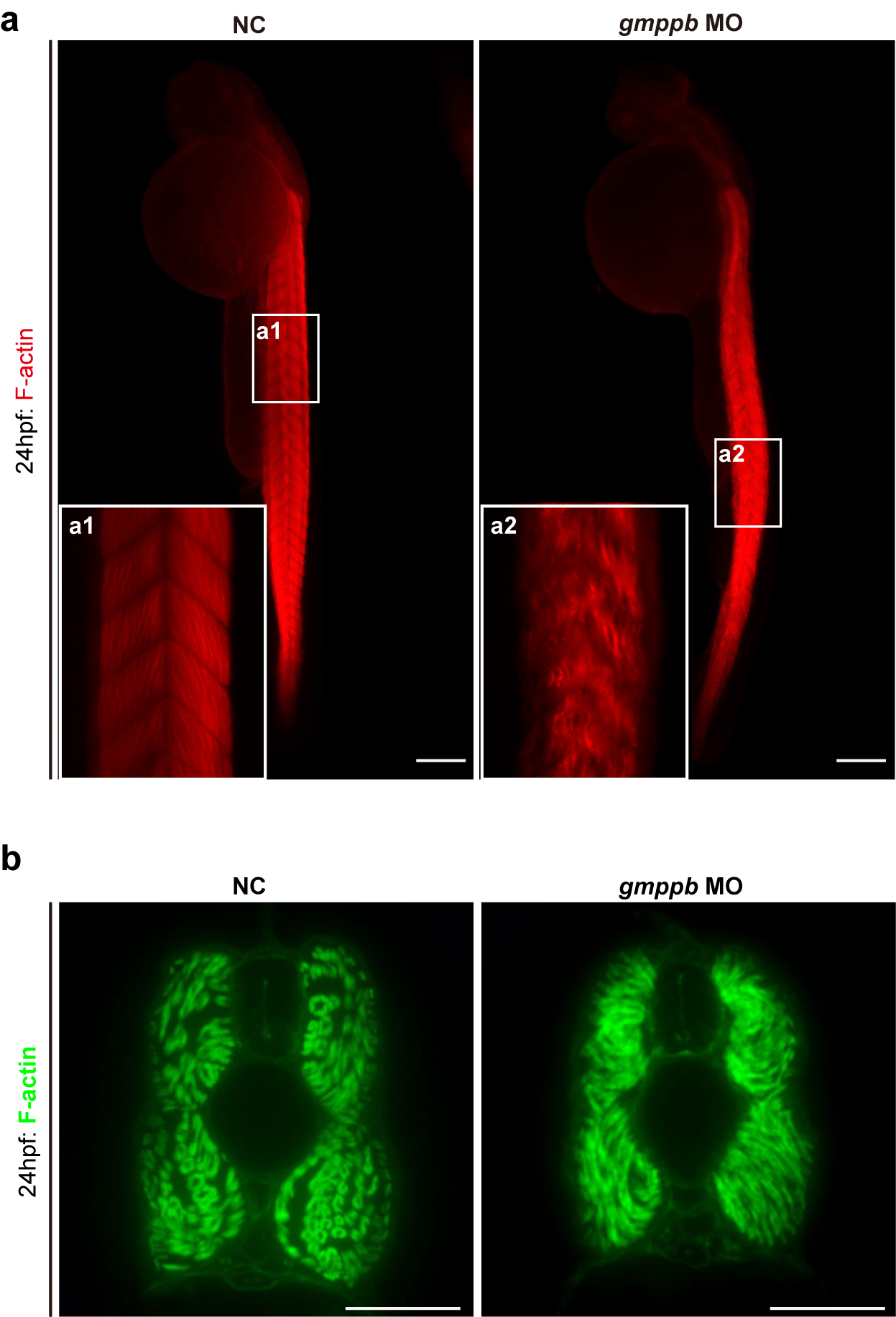


**Figure S2.** GMPPB KD causes muscle defects in zebra fish. **(a)** Phalloidin staining (red) of filamentous actin in zebra fish injected with control MO (NC), or gmppb MO at one-cell stage. Scale bar: 250 μm **(b)** Phalloidin staining (green) on the cross-sections of 24 hpf embryos injected with control MO (NC), or gmppb MO at one-cell stage. Scale bar: 100 μm


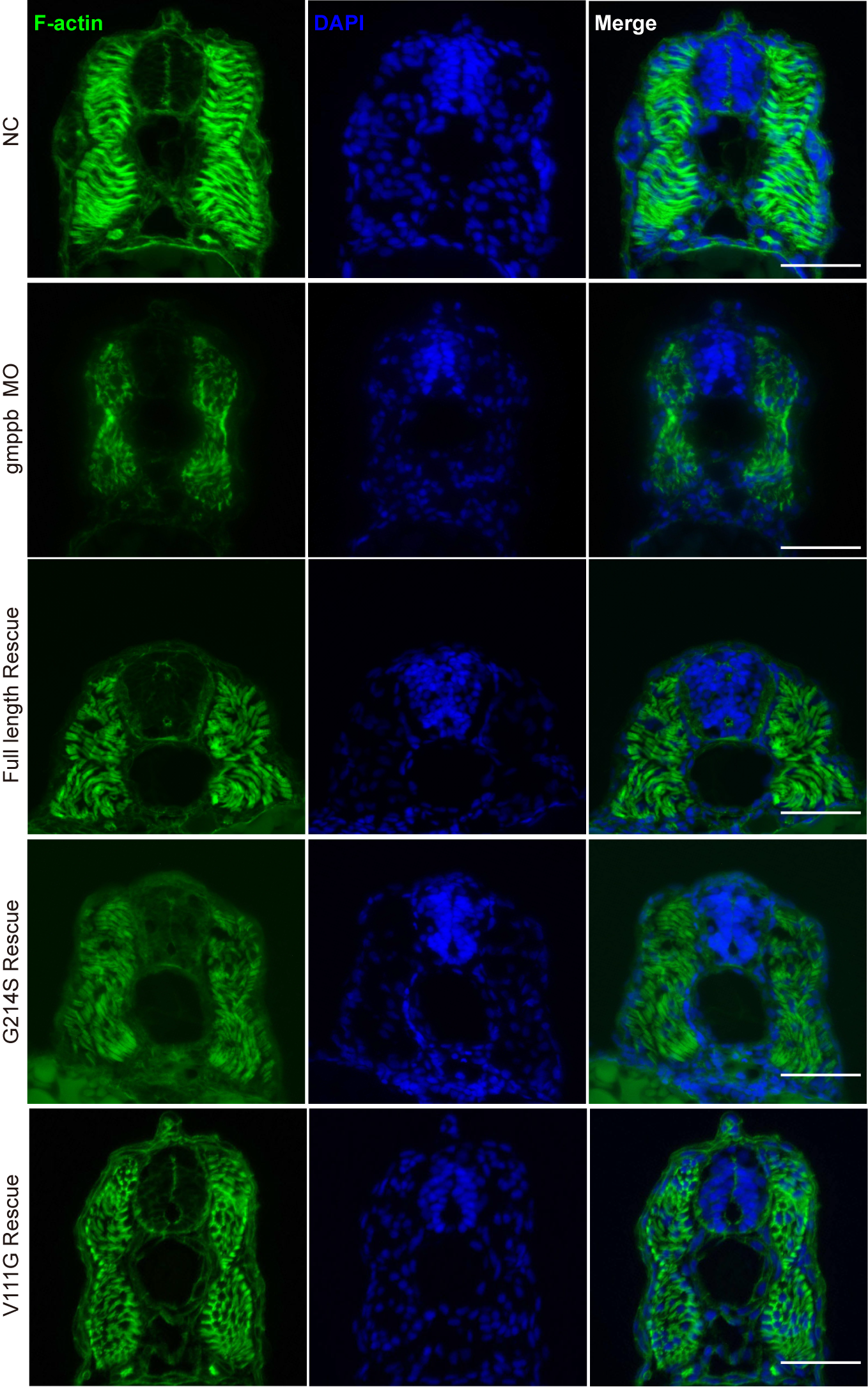


**Figure S3.** GMPPB V111G mutant fails to rescue muscle defects in zebra fish caused by GMPPB KD. Phalloidin (green) and DAPI (blue) staining on the cross-sections of 48 hpf embryos injected with control MO (NC), or gmppb MO alone or together with mRNA encoding GMPPB WT or its mutants at one-cell stage. Scale bar: 100 μm
